# MAP3K1 Integrates Genetic and Environmental Signals in Eyelid Morphogenesis

**DOI:** 10.3390/cells15171508

**Published:** 2026-08-22

**Authors:** Bo Xiao, Winston Kao, Ying Xia

**Affiliations:** 1 Department of Environmental and Public Health Sciences, College of Medicine, University of Cincinnati, 160 Panzeca Way, Cincinnati, OH 45267-0056, USA; bobo199005@163.com; 2 Department of Ophthalmology, College of Medicine, University of Cincinnati, 231 Albert Sabin Way, Cincinnati, OH 45267-0527, USA; kaoww@ucmail.uc.edu

**Keywords:** MAP3K1 signaling network, dioxin, aryl hydrocarbon receptor (AHR), genetic crosstalk, gene-environment interactions, embryonic eyelid closure

## Abstract

Developmental disorders often arise from complex interactions between genetic variation and environmental factors, yet the molecular mechanisms underlying gene-gene (G × G) and gene-environment (G × E) interactions remain poorly understood. Mouse embryonic eyelid closure provides a genetically tractable in vivo model for investigating these mechanisms. Eyelid closure requires coordinated epithelial migration and cytoskeletal remodeling orchestrated by interconnected signaling pathways. Among these pathways, MAP3K1 functions as a critical signaling hub that integrates inputs from S1PR-RHOA-ROCK and other upstream regulators to activate JNK and promote eyelid closure. Genetic studies show that multiple components within the GPCR-RHOA-ROCK-MAP3K1-JNK network cooperate to maintain developmental robustness. Reducing the activity of pathway components dose-dependently impairs eyelid closure and produces the characteristic eye-open-at-birth (EOB) phenotype. Environmental factors also converge on this network. Although exposure to dioxin does not impair eyelid closure in wild-type embryos, it induces EOB in embryos harboring otherwise phenotypically silent mutations in the MAP3K1 network, such as *Map3k1*^+/−^, *Jnk1*^−/−^ and *S1pr2*^−/−^. These findings identify the MAP3K1 pathway as a point of convergence of genetic and environmental signals and establish embryonic eyelid closure as a powerful model for elucidating molecular mechanisms underlying developmental robustness, susceptibility and resilience.

## 1. Introduction

Understanding the causes of human disease requires consideration of both genetic predisposition and environmental exposure. Advances in genome-wide association studies, whole-exome sequencing, and whole-genome sequencing have identified thousands of genetic variants associated with human diseases and developmental disorders [1,2,3,4]. These discoveries have greatly improved our ability to assess disease susceptibility and have enabled the development of models for individual and population-level risk assessment [1,2,3,4]. However, the biological impact of a genetic variant is often shaped by epistatic interactions with other variants within the broader genomic landscape. As a result, disease susceptibility is determined by the combined effects of numerous variants acting through interconnected molecular pathways.

Environmental factors constitute additional and equally important determinants of susceptibility. Throughout life, cells and tissues are continuously exposed to environmental influences, including chemical toxicants, infectious agents, nutritional conditions, psychosocial stressors, and physical stimuli [5]. Recent advances in exposome-wide association studies, high-resolution metabolomics, and large-scale epidemiological analyses have substantially expanded our ability to characterize environmental influences on health and disease [6,7,8]. Nevertheless, individuals exposed to similar environmental conditions can exhibit different biological outcomes, highlighting the role of genetic background in environmental responses.

The interplay between genetic and environmental factors is increasingly recognized as a critical determinant of complex diseases [9,10]. G × G interactions shape how combinations of genetic variants influence biological functions, whereas G × E interactions determine how environmental exposures reveal, amplify, or mitigate genetic susceptibility. Despite their importance, the molecular mechanisms underlying these interactions remain poorly understood. Rather than resulting from isolated molecular events, G × G and G × E effects likely emerge when genetic and environmental perturbations converge on interconnected signaling networks that collectively determine the capacity to maintain homeostasis. Dissecting these mechanisms requires tractable models that enable genetic and environmental perturbations to be manipulated independently and in combination, while allowing quantitative assessment of the biological outcomes.

Mouse embryonic eyelid closure provides a suitable in vivo model for studying these mechanisms. Eyelid morphogenesis is a highly coordinated process that depends on epithelial migration, cytoskeletal remodeling, and tissue fusion. Failure of this process results in the readily identifiable eye-open-at-birth (EOB) phenotype. This phenotype provides a convenient readout of developmental perturbation. Studies using this model have revealed multiple signaling pathways and genetic factors that regulate morphogenesis. They have also identified MAP3K1 as a central signaling hub integrating diverse developmental inputs.

Here we summarize current knowledge of the mechanisms governing embryonic eyelid closure, with a focus on the MAP3K1-centered signaling network. We discuss how this network integrates genetic and environmental influences and how interactions within this network influence developmental robustness and susceptibility to morphogenetic defects.

## 2. Embryonic Eyelid Closure as a Model of Developmental Robustness and Susceptibility

**Figure 1:**
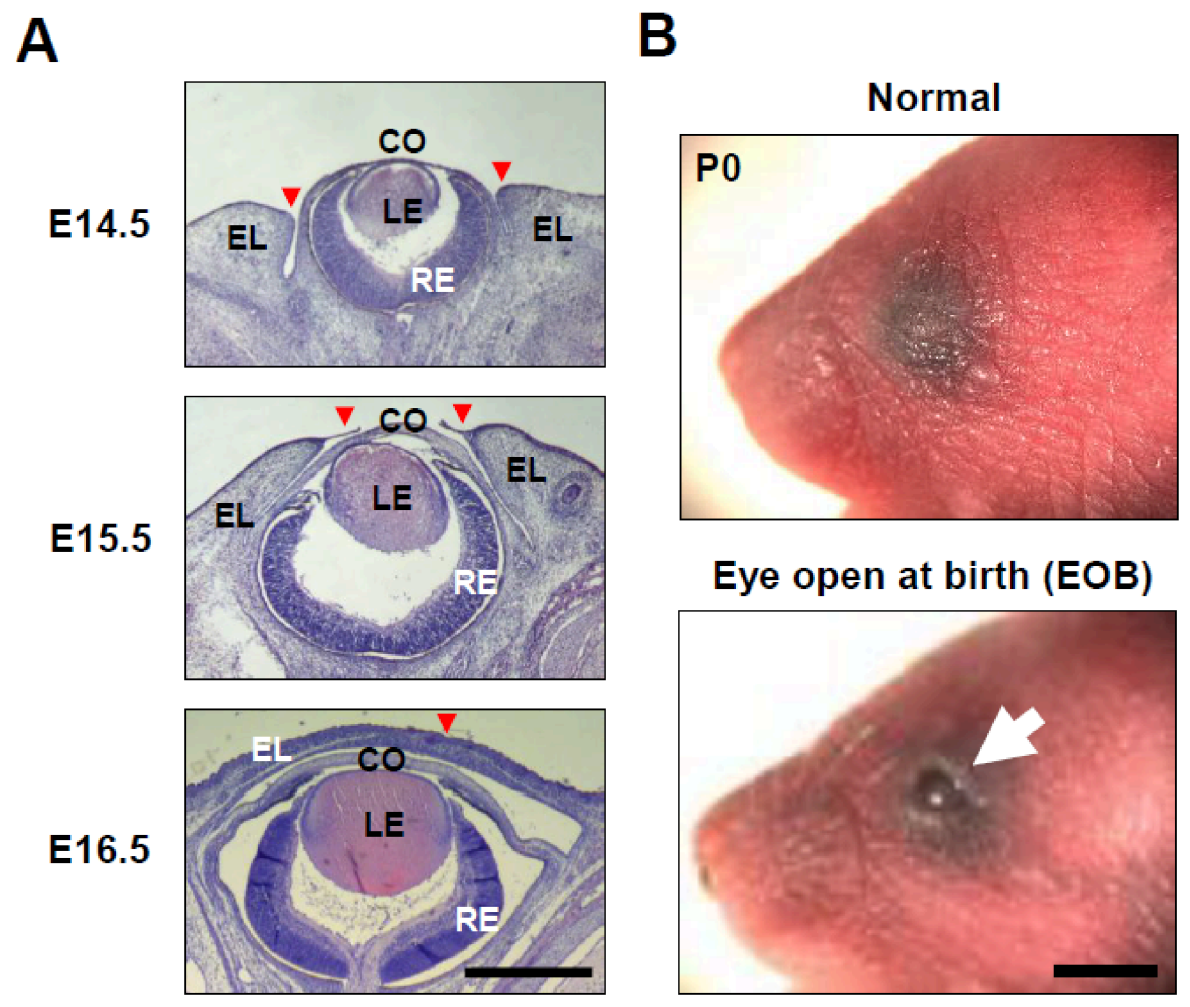
Embryonic eyelid development and the EOB phenotype. (**A**) Representative H&E-stained sections illustrating mouse eyelid morphogenesis from E14.5 to E16.5, based on previously published data [15]. During this period, the eyelid margins extend across the ocular surface and fuse at the midline. Red arrows indicate the eyelid leading edge or fusion junction. (**B**) Representative gross photographs of newborn (P0) mice, based on previously published data [16]. Normal mice are born with fused eyelids (upper panel), whereas failure of embryonic eyelid closure results in the EOB phenotype (white arrow, lower row). CO: cornea; EL: eyelid; LE: lens; RE: retina. Scale bars: 200 μm in (**A**), 500 μm in (**B**).

Transient fusion of the upper and lower eyelids during embryogenesis is a conserved feature of mammalian eye development [11]. In mice, eyelid morphogenesis begins around embryonic day 11.5 (E11.5), when the dorsal and ventral ocular surface ectoderm invaginates to form the primordial eyelid folds [12]. These folds subsequently grow and extend across the developing eye (Figure 1A). Between E15.5 and E16.0, the opposing eyelid margins meet and fuse at the midline, forming a temporary epithelial seal that protects the cornea [13]. The fused eyelids remain closed throughout late gestation and reopen approximately two weeks after birth through a developmentally programmed process involving epithelial differentiation, i.e., keratinization, and apoptosis at the fusion junction [14]. Failure of embryonic eyelid closure results in the readily identifiable and nonlethal EOB phenotype (Figure 1B).

Although eyelid closure appears morphologically simple, it requires the coordinated action of multiple cellular processes, including epithelial proliferation, migration, differentiation, cell-cell adhesion, cytoskeletal remodeling, and tissue fusion [17,18]. These processes are controlled by interconnected signaling networks. Disruption of these networks can impair eyelid closure and lead to the EOB phenotype.

## 3. Signaling Control of Embryonic Eyelid Closure

Genetic studies have identified a growing repertoire of mutations that produce the EOB phenotype (http://www.informatics.jax.org/) (Table 1). These genes encode extracellular ligands, cell-surface receptors, transcription factors, cytoskeletal regulators, and intracellular signaling molecules. Although functionally diverse, many EOB-associated genes converge on a limited number of signaling pathways, including EGF, TGFβ/BMP, FGF, GPCR, WNT/planar cell polarity (PCP), integrin, and MAPK signaling (Table 1).

### 3.1. EGFR Signaling Defines Pathway Regulation of Eyelid Morphogenesis

**Figure 2:**
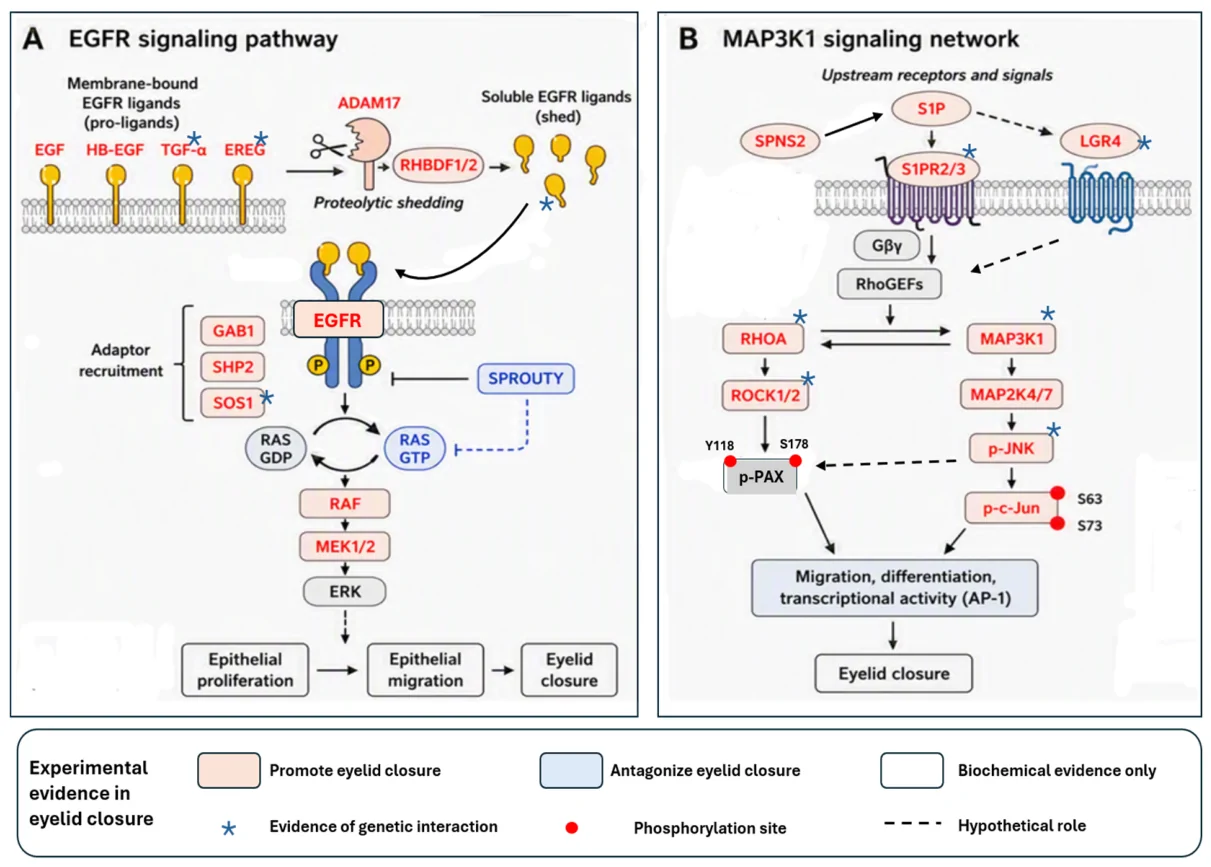
Genetic studies define signaling pathways controlling embryonic eyelid closure. (**A**) EGFR signaling pathway. RHBDF1/2-dependent activation of ADAM17 promotes proteolytic shedding of membrane-bound EGFR ligands, including EGF, HB-EGF, TGF-α, and EREG, allowing EGFR activation [19,20,21,22,89,90]. Receptor activation recruits signaling adaptors, including GAB1, SHP2, and SOS1, to initiate the downstream RAF-MEK1/2 cascade [23,41,91]. This pathway regulates epithelial proliferation, migration and morphogenetic processes required for eyelid fusion. SPROUTY and RAS are shown as negative regulators of EGFR-associated signaling [26,88]. (**B**) MAP3K1 signaling network. Extracellular S1P is transported by SPNS2 and signals through S1PR2/3 and other receptor-associated mechanisms [37,92,93]. S1PR2/3 couple to Gβγ and ARHGEFs to activate RHOA and its downstream effectors ROCK1/2, thereby regulating cytoskeletal organization and cell behavior [15,44]. LGR4 is also depicted as a receptor-associated component of the signaling network [38]. Genetic interactions between *Lgr4* and *Map3k1*, *S1pr*, and *Rhoa* mutations shown in this figure are based on unpublished data from our laboratory and are available upon request. MAP3K1 functions within this network downstream of S1PR2/3-associated signaling, downstream of RHOA-ROCK, and upstream of the MAP2K4/7–JNK cascade. Activation of MAP2K4/7 promotes JNK phosphorylation (p-JNK), followed by phosphorylation of c-Jun (p-c-Jun) and activation of AP-1-dependent transcription [15]. MAP3K1-associated signaling also regulates paxillin phosphorylation (p-PXN), linking the pathway to cell adhesion and migration. Together, these signaling pathways coordinate epithelial migration, differentiation, and transcriptional activity required for tissue morphogenesis and successful embryonic eyelid closure.

EGFR signaling provides a well-defined example of how coordinated regulation within a pathway determines developmental outcome (Figure 2A). During eyelid morphogenesis, EGFR ligands produced by the surface epithelium and adjacent mesenchyme activate EGFR in the leading-edge epithelium. This signaling promotes epithelial proliferation, directional migration, cytoskeletal remodeling, and formation of actin-rich protrusions that drive eyelid extension and fusion. Consistent with these functions, disruption of EGFR, its ligands, the ligand-shedding protease ADAM17, its regulators RHBDF1 and RHBDF2, or downstream effectors including GAB1, SHP2, RAF1, and MAP2K1/2 impairs eyelid closure and causes the EOB phenotype [20,86,87].

Genetic analyses further demonstrate that signaling output is determined by the combined activity and dosage of multiple pathway components. Deletion of an individual ligand, such as EGF, amphiregulin (AREG) and TGFα, often has little effect, whereas simultaneous reduction in multiple ligands or combined impairment of ligand and receptor activity markedly increases EOB penetrance (Table 2). Conversely, excessive pathway activation can also disrupt morphogenesis. Constitutive Ras activation or deletion of the negative regulators Sprouty1 and Sprouty2 produces EOB despite increased EGFR signaling activity [26,88]. These findings indicate that successful eyelid closure requires precise quantitative control of EGFR signaling, with both insufficient and excessive pathway activity disrupting normal development.

**Table 2:** Gene-gene interactions in mouse embryonic eyelid closure.

Signaling Pathway Functional Group	Mutants	EOB (%) Penetrance	Ref.
Canonical signaling pathways			
EGF	*Rhbdf1*^−/−^; *Rhbdf2*^−/−^	100	[89]
	*Egf*^+/−^; *Tgfa^+^*^/−^; *Areg*^+/−^	80–90	[90]
	*Spry1^ΔOSE^*^/*ΔOSE*^; *Spry2^ΔOSE^*^/*ΔOSE*^	100	[88]
	*Egfr^mut^*^/*mut*^; *Sos1*^+/−^	50	[91]
	*Egfr^ΔOSE^*^/+^; *Gab1^ΔOSE^*^/+^	0	* U
	*Egfr^ΔOSE^*^/+^; *Shp2^ΔOSE^*^/+^	0	
	*Gab1^ΔOSE^*^/+^; *Shp2^ΔOSE^*^/+^	0	
BMP	*Bmpr2^ΔEP^*^/*ΔEP*^; *Acvr2a*^−/−^	100	[94]
GPCR	*S1pr2^+^*^/−^; *S1pr3*^−/−^	0	[92,93]
	*S1pr2*^−/−^; *S1pr3*^+/−^	0	
	*S1pr2*^−/−^; *S1pr3*^−/−^	36	
	*Lgr4* ^−/−^	6	[38]
	*S1pr2*^+/−^; *S1pr3*^+/−^; *Lgr4*^−/−^	100	* U
	*S1pr2*^+/−^; *Lgr4*^−/−^	67	
MAPK	*Map3k1*^+/−^; *Jnk1*^−/−^	100	[16]
	*Map3k1*^+/−^; *Jnk2*^−/−^	0	
	*Map3k1*^+/−^; *Jnk1*^+/−^; *Jnk2*^+/−^	NR	
	*Jnk1*^−/−^; *Jnk2*^−/−^	100	[95]
	*Map3k1*^+/−^; *Jun^ΔOSE^*^/+^	0	[96]
	*Map2k1^Δep^*^/*Δep*^; *Map2k2*^−/−^	NR	[97]
WNT	*Fzd3*^−/−^; *Fzd6*^−/−^	10	[98]
	*Dkk2* ^+/−^	0	[99,100]
	*Dkk2* ^−/−^	100	
	*Wls^ΔOSE^* ^/+^	0	
	*Wls^ΔOSE^* ^/*ΔOSE*^	100	
	*Dkk2*^+/−^; *Wls^ΔOSE^*^/+^	0	
Integrin	*Itga5^TG^*^/+^; *Itgb1^TG^*^/+^	74	[57]
	*Itga2^TG^*^/+^; *Itgb1^TG^*^/+^	25	
RHOA-ROCK	*Rhoa^ΔOSE^*^/*ΔOSE*^; *Rock1^ΔOSE^*^/+^	54	[15]
	*Rhoa^ΔOSE^*^/+^; *Rock1^ΔOSE^*^/*ΔOSE*^	6	
	*Rhoa^ΔOSE^*^/*ΔOSE*^; *Rock1^ΔOSE^*^/*ΔOSE*^	95	
	*Rock1*^+/−^; *Rock2*^+/−^	100	[44]
Pathway interactions			
MAP3K1-JNK × GPCR	*S1pr2*^+/−^; *S1pr3*^+/−^; *Map3k1*^+/−^	14	[93,99]
	*S1pr2* ^−/−^ *; Map3k1* ^+/−^	30	
	*S1pr2*^−/−^; *S1pr3*^+/−^; *Map3k1*^+/−^	32	
	*S1pr2*^+/−^; *S1pr3*^−/−^; *Map3k1*^+/−^	41	
	*S1pr2*^−/−^; *S1pr3*^−/−^; *Map3k1*^+/−^	100	
	*S1pr2*^+/−^; *S1pr3*^−/−^; *Jnk1*^+/−^	5	
	*S1pr2*^+/−^; *S1pr3*^−/−^; *Jnk1*^−/−^	63	
	*S1pr2*^−/−^; *S1pr3*^−/−^; *Jnk1*^+/−^	100	
	*Map3k1*^+/−^; *Lgr4*^−/−^	100	* U
MAP3K1-JNK × RHOA-ROCK	*Map3k1*^+/−^; *Rhoa^ΔOSE^*^/*ΔOSE*^	100	[15]
	*Map3k1*^+/−^; *Rock1^ΔOSE^*^/*ΔOSE*^	77	
	*Map3k1*^+/−^; *Rhoa^ΔOSE^*^/+^; *Rock1^ΔOSE^*^/+^	31	
GPCR × RHOA-ROCK	*S1pr2*^+/−^; *Rhoa^ΔOSE^*^/*ΔOSE*^	19	
	*S1pr2*^−/−^; *Rhoa^ΔOSE^*^/*ΔOSE*^	73	
	*S1pr3*^+/−^; *Rhoa^ΔOSE^*^/*ΔOSE*^	60	
	*S1pr3*^−/−^; *Rhoa^ΔOSE^*^/*ΔOSE*^	100	
	*S1pr2*^+/−^; *Rock1^ΔOSE^*^/*ΔOSE*^	15	
	*S1pr3*^+/−^; *Rock1^ΔOSE^*^/*ΔOSE*^	0	
	*S1pr3*^−/−^; *Rock1^ΔOSE^*^/*ΔOSE*^	61	
	*Lgr4*^−/−^; *Rhoa^ΔOSE^*^/+^	90	* U
WNT × GPCR and MAP3K1	*Dkk2*^+/−^; *Map3k1*^+/−^	0	[100]
	*Wls^ΔOSE^*^/+^; *Map3k1*^+/−^	0	
	*Dkk2*^+/−^; *Lgr4*^−/−^	32	* U
LIMK × MAP3K1 and RHOA	*Limk2*^+/−^; *Map3k1*^+/−^	0	* U
	*Limk2*^+/−^; *Rhoa^ΔOSE^*^/*ΔOSE*^	0	
Transcription factors			
	*Alx3^+^*^/*–*^; *Alx4^lst–J^*^/*lst*−*J*^	NR	[101]
	*Cebpa^Δep^*^/*Δep*^; *Cebpb^Δep^*^/*Δep*^	100	[102]
	*Foxc1*^+/−^; *Foxc2*^+/−^	NR	[103]
	*Grhl3*^−/−^; *Lmo4*^−/−^	54	[73]
	*Pbx1*^−/−^; *Pbx2*^−/−^	NR	[78]
	*Smad1^ΔOSE^*^/*ΔOSE*^; *Smad5 ^ΔOSE^*^/*ΔOSE*^	100	[104]

NR = EOB observed, but incidence not reported in original publication; * U, unpublished data (Mongan, Xiao and Xia). Background shading is used to distinguish functional categories and improve table readability.

Components labeled in red boxes/texts have genetic evidence supporting a role in eyelid closure. Asterisks indicate genetic interactions within the signaling pathways. Blue boxes denote factors whose genetic alteration antagonizes eyelid closure, whereas gray boxes denote components supported by biochemical evidence but not yet genetically tested for a role in eyelid closure.

**Figure 3:**
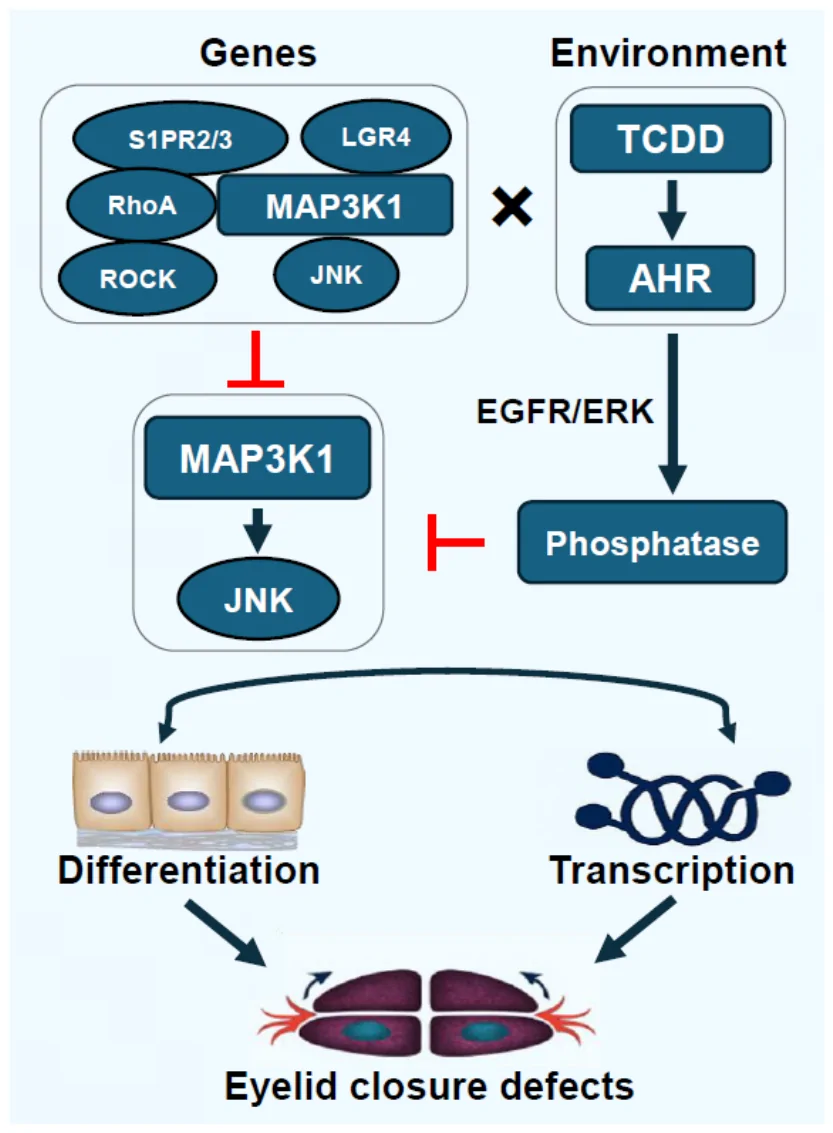
G × E interactions converge on the MAP3K1-JNK signaling axis to disrupt embryonic eyelid closure. The model illustrates how genetic variation and environmental exposure interact to alter signaling output during eyelid morphogenesis. Genetic perturbations affecting components of the MAP3K1 signaling network, including *S1pr2/3*, *Lgr4*, *Rhoa*, *Rock*, *Map3k1*, and *Jnk*, reduce the activity or capacity of the MAP3K1-JNK pathway [15,16,105]. Under normal conditions, MAP3K1 promotes JNK activation, which contributes to transcriptional programs and epithelial behaviors required for eyelid closure. Environmental exposure to the dioxin-like compound TCDD activates the aryl hydrocarbon receptor (AHR). AHR signaling increases EGFR/ERK activity and induces phosphatase activity, which suppresses MAP3K1-JNK signaling [15,93,106]. In genetically normal embryos, this reduction in signaling can remain within the range tolerated for successful eyelid closure. However, genetic perturbations that reduce MAP3K1 network activity create a sensitized state in which additional TCDD-AHR-mediated suppression can lower signaling output below the level required for normal morphogenesis. Reduced MAP3K1-JNK signaling alters epithelial differentiation and transcriptional programs [107,108], ultimately impairing the coordinated cellular behaviors required for eyelid fusion and resulting in EOB. Red inhibitory bars indicate suppression; black arrows indicate signaling or downstream effects; and the × symbol denotes interaction between genetic and environmental perturbations.

### 3.2. MAP3K1-JNK Signaling Is a Core Morphogenetic Module

Whereas EGFR signaling highlights the importance of pathway contribution, MAP3K1 signaling provides insight into how molecular inputs are integrated into a network. MAP3K1 is a serine/threonine kinase that links diverse upstream signals to MAPK signaling and coordinates transcriptional and cytoskeletal responses [109,110]. Among its developmental functions, regulation of embryonic eyelid closure is the most consistently observed. Independent mouse models, including *Map3k1*-null (*Map3k1*^−/−^), kinase-deficient (*Map3k1^Δ^^KD^*^/^*^Δ^^KD^*), and spontaneous truncation mutant (lid-gap gate, lg-Ga), all show fully penetrant EOB phenotypes [40,111,112,113,114], establishing an essential role for MAP3K1 in eyelid morphogenesis.

During eyelid development, MAP3K1 is highly expressed in the leading-edge epithelium, where it promotes JNK activation in migratory keratinocytes [40]. Activated JNK phosphorylates c-JUN and other downstream effectors to regulate gene expression, actin remodeling, cell-cell adhesion, and epithelial migration. Accordingly, MAP3K1 deficiency reduces JNK and c-JUN phosphorylation, disrupts actin organization, and impairs epithelial migration at the eyelid margin [40,108]. Disruption of JNK signaling also causes EOB, highlighting the importance of JNK signaling in eyelid morphogenesis [95].

Genetic interaction studies support functional cooperation between MAP3K1 and JNK. Neither *Map3k1* heterozygosity nor partial reduction in JNK activity alone disrupts eyelid closure; however, simultaneous reduction in both components produces EOB with variable penetrance and severity [16] (Table 2). These interactions suggest that MAP3K1 and JNK act in the same pathway and together maintain the signaling output required for eyelid closure. In contrast, although complete loss of c-JUN also causes EOB, c-JUN heterozygosity does not enhance the phenotypes of *Map3k1* or *Jnk* mutants. Thus, c-JUN dosage may not be rate-limiting for MAP3K1-JNK signaling in eyelid closure [75,93].

### 3.3. GPCR Signaling Provides Major Inputs to the MAP3K1-JNK Module

Genetic interaction studies further support a functional relationship between MAP3K1 and GPCR signaling [93] (Figure 2B, Table 2). Among these GPCRs implicated in eyelid closure, sphingosine-1-phosphate receptors (S1PRs) are the best-characterized. S1PR2 and S1PR3 function redundantly to regulate epithelial migration and tissue fusion. Simultaneous loss of both receptors results in the EOB phenotype [115] (Table 2). Deletion of SPNS2, which transports S1P to the extracellular space, also disrupts eyelid closure [37]. Thus, S1P availability and its receptors are both important for normal eyelid morphogenesis [37] (Table 1). Genetic and biochemical evidence places MAP3K1 downstream of S1PR signaling. In cultured keratinocytes, MAP3K1 deficiency attenuates S1P-induced JNK activation and epithelial migration. In vivo, partial loss of S1PR2 or S1PR3 enhances the EOB phenotype in *Map3k1* and *Jnk1* mutants [93]. These findings support a functional S1PR-MAP3K1-JNK axis that promotes epithelial migration required for eyelid closure.

Another GPCR implicated in eyelid closure is LGR4. LGR4 is primarily recognized as a receptor for R-spondins that potentiates canonical WNT signaling by stabilizing Frizzled-LRP5/6 receptor complexes and antagonizing ZNRF3/RNF43-mediated receptor turnover [116]. However, its function in eyelid morphogenesis appears to extend beyond canonical WNT signaling [117,118]. Genetic interaction studies show that *Lgr4* mutations enhance the EOB phenotypes of *Map3k1*, *S1pr*, and *Rhoa* mutants, suggesting that LGR4 contributes to the MAP3K1 network Another GPCR implicated in eyelid closure is LGR4. LGR4 is primarily recognized as a receptor for R-spondins that potentiates canonical WNT signaling by stabilizing Frizzled-LRP5/6 receptor complexes and antagonizing ZNRF3/RNF43-mediated receptor turnover [116]. However, its function during eyelid morphogenesis appears to extend beyond canonical WNT signaling [117,118]. Genetic interaction studies show that *Lgr4* mutations potentiate the EOB phenotypes of *Map3k1*, *S1pr*, and *Rhoa* mutants, suggesting that LGR4 contributes to the network that regulates eyelid morphogenesis (Table 2). While these genetic interactions place LGR4 within the MAP3K1-associated network, direct biological evidence linking LGR4 to MAP3K1 signaling remains limited.

### 3.4. RHOA-ROCK Signaling Links GPCR Activation to MAP3K1

A key intermediate linking GPCR signaling to MAP3K1 is the RHOA-ROCK pathway. RHOA is a small GTPase that regulates actomyosin contractility, focal adhesion maturation, stress fiber assembly, and cellular tension through its downstream effectors ROCK1 and ROCK2. These cytoskeletal processes generate the mechanical forces necessary for epithelial migration and tissue remodeling during morphogenesis [119,120]. Consistent with this role, combined deletion of *Rock1* and *Rock2* results in fully penetrant EOB [37,44,121] (Table 2). Likewise, RHOA deficiency alone has minimal effects on eyelid development but causes severe closure defects when combined with *Rock1* mutations. These data suggest that cumulative reduction in RHOA-ROCK signaling can compromise eyelid closure [15].

Multiple lines of evidence position RHOA-ROCK signaling within the GPCR-MAP3K1 network. Partial reduction in RHOA or ROCK activity enhances the eyelid defects associated with *S1pr2/3, Lgr4*, and *Map3k1* mutations [15,105] (Table 2). In cultured keratinocytes, S1P stimulation activates JNK, induces c-JUN phosphorylation and AP-1 transcription activity, promotes paxillin phosphorylation, and enhances directional migration. These responses require RHOA, ROCK, and MAP3K1 (Figure 2B). Conversely, MAP3K1 also contributes to RHOA-dependent focal adhesion signaling, suggesting bidirectional communication between cytoskeletal and MAPK signaling [15].

Recent proximity-labeling studies provide additional evidence for this signaling network. TurboID-based proteomic analyses identified Gβ2 and multiple Rho guanine nucleotide exchange factors (RhoGEFs) among MAP3K1-associated proteins. These proteins may link GPCR activation to both RHOA and MAP3K1 signaling [15]. Although the precise molecular connections remain to be established, these findings suggest that GPCR inputs are integrated with RHOA-ROCK and MAP3K1-JNK modules.

## 4. Defining Signaling Networks Through Genetic Interaction Studies

Genetic interaction analysis provides a powerful in vivo approach for understanding functional relationships among genes. When gene products function in the same biological pathway or form a common molecular complex, mutations in the corresponding genes can exhibit non-allelic non-complementation, in which individually mild or silent mutations produce a more severe phenotype when combined [122]. Embryonic eyelid closure provides an especially sensitive model for detecting such interactions. By testing whether combined mutations produce a stronger eyelid closure defect than either mutation alone, this model can reveal functional relationships among signaling components in vivo.

Studies using this model have uncovered extensive interactions between paralogous genes within the EGF, BMP, S1PR, ROCK, JNK and transcription factor families (Table 2). Similar interactions have been observed among components of the MAP3K1 network [15]. Combined mutations involving *S1prs*, *Lgr4*, *Rhoa*, *Rock1*, *Map3k1*, and *Jnk1* produce EOB, whereas individual mutations are largely tolerated. It is worth noting that non-allelic non-complementation is not observed between MAP3K1 network genes and genes primarily involved in EGFR or canonical WNT signaling [106].

A notable feature of these network-specific interactions is the range of developmental outcomes, from normal closure to delayed closure, incomplete fusion, and fully penetrant EOB [15,16,99,105]. Phenotypic severity correlates inversely with p-JNK levels, suggesting that progressively reduced JNK signaling contributes to increasingly severe closure defects [93]. Thus, the EOB phenotype is not merely a morphological endpoint. It provides a sensitive functional readout of signaling activity, revealing how individual genes and their interactions influence pathway output.

These observations support a conceptual threshold model of developmental robustness in which successful eyelid morphogenesis is maintained when integrated signaling output remains above a critical level. As genetic perturbations accumulate, signaling capacity may progressively decline, increasing the likelihood and severity of developmental failure [93]. Although this threshold model has not been directly demonstrated experimentally, the observed relationship between p-JNK levels and phenotypic severity provides evidence consistent with such a model. This model provides a potential explanation for how G × G interactions influence phenotypic outcomes and how environmental exposures may further reduce signaling capacity to modify developmental susceptibility through G × E interactions.

## 5. Environmental Perturbation of the MAP3K1 Network

Although embryonic eyelid closure is genetically programmed, it remains highly responsive to environmental influences. Numerous pharmaceuticals, toxicants, and maternal exposures can disrupt eyelid morphogenesis and induce EOB in mice [123,124,125,126,127,128]. This environmental sensitivity makes the eyelid closure model well suited for investigating G × E interactions. G × E interactions occur when a variant that has little or no effect under normal conditions increases susceptibility to otherwise tolerated environmental challenges. Early evidence for such interactions came from a spontaneous EOB mutant strain. Maternal administration of hormones or retinoic acid partially rescued eyelid development in these mutants [129]. Therefore, the developmental outcomes depend not only on genetic background but also on its interaction with environmental factors.

A particularly informative example of G × E regulation involves the aryl hydrocarbon receptor (AHR) pathway. Dioxin-like compounds are persistent environmental pollutants that exert their biological effects primarily through activation of the AHR, a ligand-activated transcription factor of the basic helix-loop-helix/Per-Arnt-Sim (bHLH-PAS) family [130,131]. Following ligand binding, AHR translocates to the nucleus, dimerizes with Aryl hydrocarbon receptor nuclear translocator (ARNT), and regulates transcriptional programs involved in xenobiotic metabolism, cellular differentiation, and developmental signaling [132,133,134].

Among environmental AHR ligands, 2,3,7,8-tetrachlorodibenzo-p-dioxin (TCDD) is widely used as a prototypical compound for investigating AHR-mediated developmental toxicity [135,136]. Studies using the eyelid closure model have revealed selective interactions between TCDD-AHR signaling and the MAP3K1 network. TCDD exposure alone does not impair eyelid closure in wild-type embryos. However, it dose-dependently induces EOB in embryos carrying otherwise silent mutations in *Map3k1*, *S1pr2*, *S1pr3*, *Rhoa*, *Rock1*, *Lgr4*, *and Jnk1* [15,93,106] (Figure 3, Table 3 and Table 4). In contrast, mutations in genes outside this network*,* including *Egfr*, *Gab1*, *Shp2*, *Dkk2*, and *Limk2*, do not confer similar sensitivity to TCDD. These findings indicate that TCDD does not broadly impair eyelid development but preferentially disrupts a developmental program that depends on the MAP3K1 signaling module.

Several lines of evidence establish AHR activation as the initiating event in this response. TCDD strongly induces canonical AHR target genes in the developing eyelid epithelium, and deletion of *Ahr* largely abolishes TCDD-induced EOB in genetically sensitized embryos [93]. Furthermore, susceptibility correlates closely with AHR ligand-binding affinity, with embryos carrying high-affinity receptor alleles responding to substantially lower doses of TCDD [15,106].

AHR activation subsequently perturbs signaling pathways that regulate morphogenesis (Figure 3). A prominent TCDD-responsive pathway is EGFR signaling. TCDD induces EGFR ligand expression and prolongs ERK activation, which leads to suppression of JNK-dependent signaling [93]. This reduction is tolerated in genetically normal embryos but becomes consequential when MAP3K1 pathway activity is already reduced by genetic perturbation. In sensitized embryos, TCDD-induced activation of EGFR-ERK signaling further reduces JNK pathway output, potentially lowering it below the threshold required for epithelial migration and tissue fusion and thereby causing eyelid closure failure [15,106]. Consistent with this mechanism, reducing EGFR signaling by deleting one *Egfr* allele significantly attenuates TCDD-induced EOB in *Map3k1* heterozygous embryos [93].

These signaling alterations are accompanied by changed epithelial behavior. TCDD increases expression of epithelial differentiation markers, i.e., *Krt1* and *Krt10*, while suppressing migration-associated programs, particularly in *Map3k1*- and *Rhoa*-deficiency conditions [15,93]. Thus, environmental exposures can unmask otherwise silent genetic vulnerabilities by further reducing signaling output below the level required for successful morphogenesis.

## 6. Perspectives and Future Directions

Studies of embryonic eyelid closure have established MAP3K1 as a key regulator of epithelial morphogenesis. Genetic and molecular studies have positioned MAP3K1 within a signaling network that links GPCR, RHOA-ROCK, and JNK pathways to the cellular behaviors required for eyelid closure. Studies of this network further show that genetic perturbations and environmental exposure can converge on signaling output to determine developmental outcome. The eyelid closure model thus provides a tractable system for understanding how G × G and G × E interactions increase susceptibility to developmental failure.

Despite these advances, the organization of the MAP3K1 network remains incompletely defined. Critical questions include how upstream receptors and cytoskeletal cues converge on MAP3K1, how its activity is spatially and temporally regulated during morphogenesis, and how it produces specific responses among related MAPK pathways. Addressing these questions will require combining mouse genetics with quantitative imaging, proteomics, single-cell technologies, and network analyses.

New technologies provide powerful tools to define MAP3K1 signaling in vivo. A Cre-inducible TurboID-tagged MAP3K1 mouse model enables cell type-specific proximity labeling and detection of signaling complexes in their native cell context [137,138]. Initial in vitro studies have identified heterotrimeric G-protein components, RHO guanine nucleotide exchange factors, focal adhesion proteins, and actomyosin regulators among MAP3K1-associated proteins [15]. These findings explain connections between MAP3K1, GPCR signaling, cytoskeletal regulation, and MAPK activation Integrating proximity proteomics with genetic and functional studies should help define the organization and function of this network during morphogenesis.

An important next step is to determine how MAP3K1 couples mechanical forces to intracellular signaling. RHOA-dependent contractility, focal adhesion remodeling, and tissue mechanics appear to influence MAP3K1 activity. However, how these mechanical inputs are sensed and converted into MAP3K1 signaling remains largely unknown. Resolving these mechanisms could establish MAP3K1 as a critical mechanochemical integrator that links physical forces to intracellular signaling and developmental gene regulation.

The eyelid closure model also offers a unique platform for investigating environmental susceptibility at the level of signaling networks. Studies with TCDD show that environmental exposures can selectively disrupt genetically sensitized pathways rather than broadly impairing development. This approach can be extended to additional AHR ligands, including polychlorinated biphenyls, polycyclic aromatic hydrocarbons, and emerging environmental contaminants. Systematic analysis of how these exposures affect EGFR, GPCR, WNT, TGFβ/BMP pathways may reveal broader mechanisms underlying environmentally induced developmental disorders.

A major challenge will be translating these discoveries to human biology. Although eyelid fusion occurs transiently during human development and primary eyelid closure defects are uncommon, disruption of the pathways controlling this process may contribute to a broader spectrum of ocular and craniofacial abnormalities [139]. Moreover, human genetic studies have implicated MAP3K1 in disorders of sex development, hearing impairment, cancer, and tissue remodeling [140,141,142,143]. Thus, mechanisms uncovered through eyelid morphogenesis may have relevance beyond this model. They may help to explain how MAP3K1 integrates genetic variation, environmental influences, and cellular context to influence development and disease outcomes.

In summary, embryonic eyelid closure in mice has evolved from a classical developmental phenotype into a powerful in vivo model for dissecting signaling network function. Research in this model has revealed principles of pathway integration, developmental robustness, and environmental sensitivity. Continued integration of genetic, proteomic, imaging, and systems-level approaches should further define how signaling networks control tissue morphogenesis and shape susceptibility to developmental and acquired diseases.

## Figures and Tables

**Table 1:** Genetic regulators of mouse embryonic eyelid development.

	Gene	Mouse Chr	Biological Function	Genetic Alterations	Developmental Phenotype	Ref.
Growth factor signaling						
EGF	*Adam17*	12	Ligand activation	T265M substitution	EOB; wavy hair; skin defects; growth retardation; reduced viability	[19]
	*Hbegf*	18	Ligand	Conventional knockout	Delayed eyelid closure; defective epithelial cell migration; reduced actin cytoskeleton remodeling	[20]
	*Tgfa*	6	Ligand	Conventional knockout	EOB; wavy hair; defective meibomian glands; hair follicle abnormalities; reduced epithelial proliferation	[21]
	*Egfr*	11	Receptor	Conventional knockout	EOB; perinatal lethality; epithelial immaturity; multiorgan failure; growth retardation; skin and hair defects	[22]
	*Gab1*	8	Adaptor	PI3K-docking mutant	Incomplete penetrant EOB; impaired wound closure induced by TGFα	[23]
	*Dgkd*	1	Kinase	Conventional knockout	EOB; perinatal lethality	[24]
	*Ptpn11*	2	Phosphatase	Chimeric knockout	Incomplete penetrant EOB; neonatal/perinatal lethality; abnormal limb development	[25]
	*Ras*	Tg	Small GTPase	H-Ras (G12V) OSE-overexpression	Defective embryonic eyelid closure; lens and corneal developmental defects; increased proliferation and apoptosis	[26]
FGF	*Fgf10*	13	Ligand	Conventional knockout	EOB; perinatal lethality; perturbed limb and lung development	[27]
	*Fgfr2*	7	Receptor	OSE-specific knockout	EOB; small lenses	[28]
TGFβ/BMP	*Inhbb*	2	Ligand	Conventional knockout	Incomplete penetrant EOB; female reproductive failure	[29]
	*Bmpr1a*	12	Receptor	K14-specific knockout	EOB; limb defects; perinatal lethality	[30]
	*Smad4*	18	Nuclear factor	OSE-specific knockout	EOB; microphthalmia; cataract; abnormal cell proliferation and apoptosis	[31]
Cell polarity & morphogenesis						
WNT/PCP	*Celsr1*	15	Cadherin/receptor	Missense mutation	Delayed or failure of eyelid closure; defects in hair cells in the organ of Corti; Neural tube defects	[32]
	*Ptk7*	4	Co-receptor	Conventional knockout	Open eyelids; craniorachischisis; perinatal lethality; neural tube closure defect; other abnormalities	[33]
	*Scrib*	15	PCP protein	Conventional knockout	EOB; neural tube closure defect; perinatal lethality; gonad morphological abnormalities; incomplete penetrant abdominal wall closure defect	[34]
	*Prickle1*	15	PCP protein	Gene-trap allele (hypomorphic)	EOB; shortened limbs and snout; cleft palate; curly tail; hair orientation defect	[35]
	*Vangl2*	1	PCP protein	TM domain deletion	Some showed delayed or failure of eyelid closure; mild tail loops or kinks; spina bifida	[36]
GPCR/S1P	*Spns2*	11	Ligand transporter	Insertional mutation	EOB	[37]
	*Lgr4*	14	Receptor	K14-specific knockout	EOB	[38]
Hippo	*Yap1*	9	Transcription factor	OSE-specific knockout	EOB	* U
MAPK and cytoskeletal signaling						
MAPK	*Map3k1*	13	MAP3K	Conventional knockout or C-terminal truncation mutant	EOB; imperforate vagina and female infertility; hearing loss	[39,40]
	*Raf1*	6	MAP3K	Conventional knockout	EOB; severe growth retardation; embryonic/perinatal lethality; skin and lung abnormalities; reduced proliferation	[41]
	*Map2k7*	8	MAP2K	K5-specific knockout	EOB; accelerated lung tumorigenesis, impaired oncogene-induced senescence	[42]
Rho/ROCK	*Rock1*	18	Kinase	Conventional knockout	EOB; ventral body wall closure defect; perinatal lethality	[43]
	*Rock2*	12	Kinase	Conventional knockout	EOB; omphalocele	[44]
	*Limk2*	1	Kinase	Conventional knockout	EOB	[45]
Signal Modulation						
Ubiquitin-proteosome	*Fbxo11*	1	E3 ubiquitin ligase	Q491L mutation	EOB; cleft palate; perinatal; Perinatal lethality	[46]
	*Fbxw7*	3	E3 ubiquitin ligase	R482Q mutation	Incomplete penetrant EOB and palate fusion; perinatal lethality	[47]
Protein phosphatases	*Ppp1r13L*	7	Phosphatase regulator	C-terminal truncation mutation	EOB; wavy fur	[48]
	*Pten*	15	Phosphatase	CNS-specific deletion	EOB; Perinatal lethality	[49]
Other kinases	*Abl1*	2	Kinase	C-terminal deletion	Incomplete penetrant EOB; smaller; postnatal lethality	[50]
	*Ikka*	10	Kinase	Conventional knockout	EOB; limb defect; perinatal lethality	[51]
	*Plk2*	8	Kinase	Conventional knockout	Incomplete penetrant EOB	[52]
Developmental metabolism						
Retinoic acid metabolism	*Cyp26b1*	5	RA metabolism enzyme	Conventional knockout	EOB; meromelia and micrognathia; limb abnormalities; perinatal lethality	[53]
	*Dhrs3*	8	RA reductase	Conventional knockout	EOB; cleft palate; perinatal lethality	[54]
Cell adhesion & epithelial integrity						
Cell adhesion	*Dsc1*	18	Desmosome	Conventional knockout	Incomplete penetrant EOB; fragile and hyperproliferative epidermis	[55]
	*Nectin1*	13	Cell adhesion	Conventional knockout	Incomplete penetrant EOB; microphthalmia	[56]
	*Itga5*	Tg	Integrin	Transgene overexpression	Incomplete penetrant EOB; abnormalities of the hairs of the coat and whiskers; skin inflammation	[57]
	*Itgb1*	Tg	Integrin	Transgene overexpression		
Genome maintenance & epigenetics						
DNA repair	*Rad23b*	4	DNA repair factor	Conventional knockout	Incomplete penetrant eyelid closure defect; growth retardation; perinatal lethality	[58]
DNA methylation	*Dnmt1*	9	DNA methylation	OSE-specific knockout	EOB and ocular abnormalities	[59]
Chromatin organization	*Hmgb1*	5	Chromatin architecture	Conventional knockout	Incomplete penetrant EOB; lung atelectasis; hypoglycemia; perinatal lethality	[60]
Epigenetic regulators	*Ahdc1*	4	Epigenetic regulator	Mosaic knockout mutant	EOB or missing eye; variable phenotypes; prenatal lethality; craniofacial abnormalities	[61]
	*Ankrd11*	8	Epigenetic regulator	Neural Crest-specific knockout	EOB; die at birth; midfacial hypoplasia; cleft palate	[62]
	*Mau2*	7	Epigenetic regulator	Neural Crest-specific knockout	EOB; facial hypoplasia; low-set ears; skull and jaw malformation	[63]
	*Nipbl*	15	Epigenetic regulator	Neural Crest-specific knockout		
Transcription regulation						
	*Alx1*	10	Transcription factor	Truncation mutant	Incomplete penetrant unilateral EOB; optical stalk disruption; cleft palate; nasal cartilage defect; perinatal lethality	[64]
	*Alx4*	2	Transcription factor	Conventional knockout	EOB; multiple bone and brain defects	[65]
	*Barx2*	9	Transcription factor	C-terminal truncation mutant	Incomplete penetrant EOB; short whiskers	[66]
	*Bcl11b*	12	Transcription factor	Conventional knockout	EOB; perinatal lethality	[67]
	*Cbfb*	8	Transcription co-factor	Endothelial-rescue of conventional knockout	Delayed or incomplete eyelid closure; skeletal defects; die at birth	[68]
	*Eya1*	1	Transcription factor	Conventional knockout	EOB; reduced head size; craniofacial and skeletal defects; absence of thymus, parathyroid glands, ears and kidneys; die at birth	[69]
	*Foxd3*	1	Transcription factor	Neural Crest-specific knockout	EOB; cleft palate; perinatal lethality	[70]
	*Foxl2*	9	Transcription factor	Conventional knockout	EOB (upper eyelid absent); female genitalia and gonad anomalies; female infertility	[71]
	*Gli3*	13	Transcription factor	Conventional knockout	Incomplete penetrant EOB; cleft palate, exencephaly; extra digit; perinatal lethality	[72]
	*Grhl3*	11	Transcription factor	Conventional knockout	Incomplete penetrant EOB; neural tube closure defects; epithelial permeability defect; perinatal lethality	[73]
	*Jun*	4	Transcription factor	K14-specific knockout	EOB	[74,75]
	*Lmo4*	5	Transcription factor	Conventional knockout	Incomplete penetrant EOB; neural tube closure defects	[73]
	*Nfib*	8	Transcription factor	DNA-binding domain mutant	EOB; perinatal lethality; delayed lung development	[76]
	*Osr2*	1	Transcription factor	Conventional knockout	EOB; cleft palate; perinatal lethality	[77]
	*Pbx1*	1	Transcription factor	R184P knock-in	EOB; subcutaneous edema; hunched posture; umbilical hernia; die at birth	[78]
	*Runx2*	17	Transcription factor	Conventional knockout	Incomplete penetrant EOB; cleft palate; molar developmental defects	[79]
	*Six1*	12	Transcription factor	Conventional knockout	Incomplete penetrant EOB; micrognathia; perinatal lethality; defects in ears, nose, kidneys and skeletal muscles	[80]
	*Ski*	5	Transcription co-repressor	Conventional knockout	Incomplete penetrant EOB; exencephaly; abnormal jaw and snout; facial cleft	[81]
	*Sox11*	12	Transcription factor	Conventional knockout	EOB; incomplete cleft palate; die after birth	[82]
	*Tbx10*	9	Transcription factor	Insertion mutation transgene overexpression	EOB; cleft palate	[83]
	*Zfp462*	4	Transcription repressor	Conventional knockout	Open eyes; neural tube defects; prenatal lethality	[84]
	*Zfp750*	11	Transcription factor	Conventional knockout	EOB; skin barrier defect; perinatal lethality	[85]

* U, unpublished data (Mongan, Xiao and Xia). Background shading is used to distinguish functional categories and improve table readability.

**Table 3:** G × E interactions in EOB (50 μg/kg TCDD).

Gene	EOB %
MAP3K1 pathway	
*Map3k1* ^+/−^	100
*Jnk1* ^+/−^	62.5
*Jnk1* ^−/−^	100
*S1pr2*^+/−^; *S1pr3*^+/−^	11.1
*S1pr2*^+/−^; *S1pr3*^−/−^	79.2
*S1pr2*^−/−^; *S1pr3*^+/−^	100
*Lgr4* ^−/−^	100
*Rhoa^ΔOSE^* ^/+^	75
*Rhoa^ΔOSE^* ^/*ΔOSE*^	100
*Rock1^ΔOSE^* ^/+^	31.3
*Rock1^ΔOSE^* ^/*ΔOSE*^	100
AHR dependency	
*Map3k1*^+/−^; *Ahr^ΔOSE^*^/*ΔOSE*^	0
*Rhoa^ΔOSE^*^/*ΔOSE*^; *Ahr*^−/−^	0
Other pathways	
*Egfr^ΔOSE^* ^/+^	0
*Dkk2* ^+/−^	0
*Gab1^ΔOSE^* ^/+^	0
*Shp2^ΔOSE^* ^/+^	0
*Limk2* ^+/−^	0

**Table 4:** TCDD-dose dependent EOB (%) incidence.

Genotype	TCDD Dose (µg/kg)			
	5	10	25	50
*Rhoa^ΔOSE^* ^/*ΔOSE*^	25.0	90.0	100	100
*Rock1^ΔOSE^* ^/*ΔOSE*^	0	50.0	75.0	100
*Rhoa^ΔOSE^*^/+^; *Rock1^ΔOSE^*^/+^	NT	58.3	NT	100

NT, not tested.

## Data Availability

No new data were created or analyzed for this review. The article includes unpublished genetic findings from our laboratory. These data are available from the corresponding author upon reasonable request.

## Abbreviation

Abbreviation	Full Term
ADAM17	A disintegrin and metalloproteinase 17
AHR	Aryl hydrocarbon receptor
AP-1	Activator protein 1
ARNT	Aryl hydrocarbon receptor nuclear translocator
CYP1A1	Cytochrome P450 family 1 subfamily a member 1
DKK2	Dickkopf-related protein 2
DLCs	Dioxin-like compounds
E11.5	Embryonic day 11.5
EGF	Epidermal growth factor
EGFR	Epidermal growth factor receptor
EOB	Eye-open at birth
ERK	Extracellular signal-regulated kinase
GAB1	GRB2-associated-binding protein 1
G × E	Gene-environment interactions
G × G	Gene-gene interactions
GPCR	G protein-coupled receptor
GWAS	Genome-wide association studies
JNK	c-JUN N-terminal kinase
KRT1	Keratin 1
LRG4	Leucine-rich repeat-containing G protein-coupled receptor 4
MAPK	Mitogen-activated protein kinase
MAP2K	Mitogen-activated protein kinasekinase
MAP3K1	Mitogen-activated protein kinase kinase kinase 1
OSE	Ocular surface epithelium
P12	Postnatal day 12
PAHs	Polycyclic aromatic hydrocarbons
PCBs	Polychlorinated biphenyls
PCP	Planar cell polarity
RHBDF2	Rhomboid 5 Homolog 2
RHOA	Ras homolog family member A
RhoGEFs	Rho guanine nucleotide exchange factors
ROCK	Rho-associated coiled-coil containing protein kinase
RSPO1-4	R-spondins
S1P	Sphingosine-1 phosphate
S1PR	Sphingosine-1 phosphate receptor
SHP2	Src homology region 2-containing protein tyrosine phosphatase 2
SPNS2	Spinster homolog 2
TCDD	2,3,7,8-Tetrachlorodibenzo-p-dioxin
TGFβ	Transforming growth factor-β
